# A Survey of Genetic Counseling for Non-invasive Prenatal Testing Examinees in Akita

**DOI:** 10.31662/jmaj.2022-0030

**Published:** 2022-06-17

**Authors:** Yohei Onodera, Hiroshi Miura, Akiko Fujishima, Rie Notomi, Atsuko Noguchi, Ikuko Takahashi, Akira Sato, Tsutomu Takahashi, Yukihiro Terada

**Affiliations:** 1 Department of Obstetrics & Gynecology, Akita University School of Medicine, Akita, Japan; 2 Department of Pediatrics, Akita University School of Medicine, Akita, Japan; 3 Department of Obstetrics, Akita Red Cross Hospital, Akita, Japan

**Keywords:** Noninvasive Prenatal Testing, Genetic Counseling, Prenatal Diagnosis

## Abstract

**Introduction:**

In recent years, there has been an increase in noninvasive prenatal testing (NIPT), where maternal blood samples are used to extract fetal cell-free DNA. Despite this being offered in several facilities in urban areas, NIPT remains to be scarcely unavailable in rural areas. Moreover, there is lacking information with regard to how pregnant women in rural areas perceive NIPT. Thus, in this study, we conducted a survey among pregnant women who came to our clinic for NIPT and examined their views on NIPT and genetic counseling.

**Methods:**

A questionnaire survey was administered to pregnant women who requested NIPT and underwent genetic counseling at our hospital between November 2016 and February 2020. The questionnaire was administered twice, once after completing the genetic counseling and once after explaining the NIPT results. The number of genetic counseling and NIPT sessions and positive test results, as well as anxiety about the test and evaluation of genetic counseling and NIPT, were assessed.

**Results:**

In total, 115 patients received genetic counseling, of which 109 underwent NIPT. The test results were found to be positive in six patients. As per our findings, 103 patients (93%) indicated they needed genetic counseling for NIPT, whereas 99 (93%) were satisfied with the counseling they received from a genetic medicine specialist. On the other hand, 82 patients (77%) requested for more testing facilities.

**Conclusions:**

The enhancement of genetic counseling systems is essential to expand the environment in which NIPT is performed. Therefore, we need to consider the need to make the NIPT testing environment more conducive and inform clients of the importance of genetic counseling in NIPT.

## Introduction

Maternal plasma is known to contain fetal DNA fragments derived from the placenta. Noninvasive prenatal testing (NIPT) uses maternal blood samples to examine the chromosomes of these DNA fragments to determine whether the fetus has chromosomal abnormalities. It is possible to diagnose aneuploidy by utilizing the massively parallel sequencing method, which comprehensively sequences cell-free DNA fragments in maternal plasma. An increase in NIPT has also been reported to reduce the number of invasive diagnoses, including amniocentesis ^(1)^.

In Japan, NIPT began in 2013 as a clinical study by the NIPT Consortium and others, which targeted high-risk pregnant women: (1) fetal ultrasound examination suggests that the fetus may have a chromosomal numerical abnormality, (2) maternal serum marker examination suggests that the fetus may have a chromosomal numerical abnormality, (3) women with a history of pregnancy with a child with a chromosome number abnormality, (4) advanced maternal age, and (5) one of the parents has a balanced Robertsonian translocation, suggesting the possibility that the fetus has 13 or 21 trisomies. Since then, the number of tests has increased with the increase in the number of testing facilities. To date, 108 facilities have been registered as accredited by the Japanese Association of Medical Sciences ^(2)^. However, there are regional differences in terms of the number of testing facilities, with some prefectures having no facilities that can perform the test, while Tokyo has 21 registered facilities. In Akita prefecture, Akita University Hospital is the only facility that can perform NIPT. Akita prefecture is located in the northeastern region of Japan and is known for its heavy snowfall in winter. It is the sixth largest prefecture in Japan. Regional and environmental differences in testing facilities have also been determined to result in differences in terms of accessibility, which may make it difficult for pregnant women living in rural areas to undergo NIPT.

Through genetic counseling, pregnant women are expected to receive accurate information, fully understand the contents of the test, and make their own decisions. Furthermore, the characteristics of pregnant women interested in genetic counseling have been reported ^(3)^. However, it remains unclear what pregnant Japanese women living in rural areas think about preNIPT genetic counseling. Therefore, in this study, we conducted a questionnaire survey among pregnant women who visited our clinic for NIPT. This study has aimed to evaluate how pregnant women living in rural areas perceive genetic counseling and NIPT and to further determine genetic testing needs.

## Materials and Methods

This study included pregnant women who visited the division of genetic counseling at Akita University Hospital and were willing to undergo NIPT between November 2016 and February 2020. The criteria for NIPT approval were as follows: pregnant women (1) with a history of pregnancy and delivery of children with chromosomal diseases (21, 18, 13 trisomies), (2) women aged ≥35 years old at the time of delivery, and (3) those who indicated that their fetus might have a chromosomal disease (21, 18, 13 trisomies). Pregnant women who wished to undergo NIPT consulted with their doctors by making appointments for consultations; some did consultations via telephone. NIPT was outsourced to GeneTech and performed by the MPS method. We analyzed the number of genetic counseling and NIPT sessions, positive test results, and the results of the survey administered after genetic counseling.

The questionnaire survey consisted of anonymous selective and open-ended responses. The questionnaire was administered twice. The first questionnaire was administered after preNIPT genetic counseling, and the second questionnaire was administered after the NIPT results were disclosed. In the first questionnaire, patients were assessed on their maternal background (maternal age, weeks of pregnancy, fertility treatment, reasons for undergoing NIPT, and access to NIPT knowledge), anxiety about NIPT before counseling, counseling evaluation, and understanding of NIPT (Table 1). The level of anxiety was defined as a 5-point scale from “very anxious” to “no anxiety” as a choice response. The counseling was rated on a 5-point scale from “sufficient” to “insufficient” using a choice-type response. After the NIPT results were explained, participants were asked to rate genetic counseling and NIPT as a second questionnaire (Table 2). The evaluation for each question was a 5-point scale from “agree strongly” to “disagree strongly” as a choice response. Genetic counseling was conducted for approximately 30 minutes per case. After genetic counseling, participants were asked about their desire to undergo NIPT. The content of genetic counseling primarily concerned chromosomal abnormalities, interpretation of positive/negative/withheld results, NIPT as a nondefinitive test, and definitive testing. Genetic counseling was provided by a pediatrician or obstetrician/gynecologist involved in genetic care. The total cost of genetic counseling and NIPT was approximately 180,000 yen.

**Table 1.** The Questionnaire after Pre-noninvasive Prenatal Testing Genetic Counseling.

Questions
Please list your age.
Please list the current number of weeks of pregnancy.
Please describe your pregnancy history.
Please select the method of conception; spontaneous conception, timed intercourse, induction of ovulation, in vitro fertilization, and intracytoplasmic sperm injection, among others.
Please describe how you came to know about NIPT; internet, newspaper or television, information from medical staff, recommendation from acquaintances, other.
Multiple choices available.
Please select your level of anxiety about NIPT before receiving genetic counseling. (5-point scale from “very anxious” to “no anxiety”).
Please rate your satisfaction with our genetic counseling: information volume, quantity of time. (5-point scale from “sufficient” to “insufficient”).
Please select your level of anxiety about NIPT after receiving genetic counseling. (5-point scale from “quite anxious” to “quite relieved”).

NIPT: noninvasive prenatal testing

**Table 2.** The Questionnaire after Disclosure of Noninvasive Prenatal Testing Results.

Questions
Please select the things that influenced for requesting NIPT; advanced maternal age, ultrasound finding, maternal serum markers, history of pregnancy, family history, family recommendation, doctor recommendation.
Multiple choices available.
Please select the statement that comes closest to your opinion on the following statement about genetic counseling. (5-point scale from “agree strongly” to “disagree strongly”).
It was worth it to get counseling from a genetic medicine specialist. It was an opportunity to think carefully about the ethical aspects of the event. Genetic counseling is necessary for NIPT. Genetic counseling should be provided by a specialist.
Please select the statement that comes closest to your opinion on the following statement about NIPT. (5-point scale from “agree strongly” to “disagree strongly”).	
Many people should know about NIPT. It is better to be able to perform NIPT at any medical institution. People with low risk of chromosomal abnormalities should also be able to undergo NIPT. The cost of NIPT is too high.

NIPT: noninvasive prenatal testing

Written informed consents were obtained from each participant. This study was approved by Ethics Review Committee of Akita University School of Medicine (reception number: 1432).

## Results

The number of patients who underwent genetic counseling for NIPT was 115, wherein 6 patients (5.2%) canceled NIPT after genetic counseling. On the other hand, the number of NIPT performed was 109. Of these, six (5.5%) tested positive.

**Figure 1.**
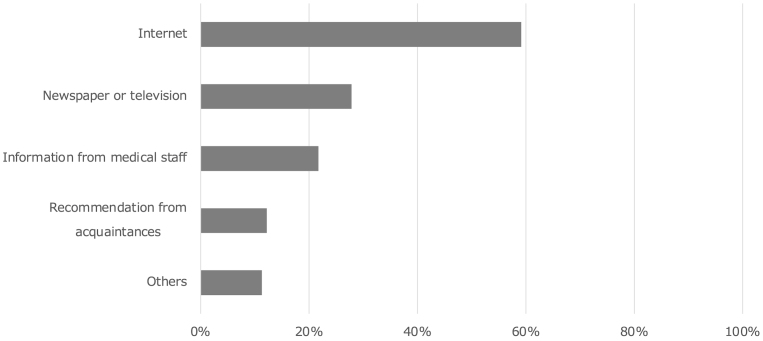
The percentage of “opportunity to know about noninvasive prenatal testing” (multiple answers available). More than half of the respondents indicated the internet as their source of information.

The mean age of patients who requested genetic counseling was 38 years. The most common reason for requesting NIPT was advanced maternal age (88%) (Table 3). The most common source of information on NIPT was the Internet (59%), followed by newspapers/television 28%, information from medical staff 22%, and recommendations from acquaintances 12% (Figure 1).

**Table 3.** Demographic and Background Characteristics of Study Participants.

Charasteristics	Number	% of all
Age at questionnaire (years)		
<35	7	6.1%
35-37	31	27.2%
38-40	51	44.7%
41-43	21	18.4%
>44	4	3.5%
Gestational age at questionnaire (weeks)		
<10	3	2.6%
10-12	69	60.5%
13-15	38	33.3%
>15	4	3.5%
Parity		
Nulliparous	51	44.7%
Parous	61	53.5%
Method of conception
Natural conseption	82	71.9%
In vitro fertilization	24	21.0%
Induction of ovulation	5	4.4%
Intracytoplasmic sperm injection	4	3.5%
Others	7	6.1%
Reason for visit (Multiple answers possible)		
Advanced maternal age	100	87.7%
Family’s recommendation	26	22.8%
Doctor’s recommendation	6	5.3%
Abnormal sonographic findings or markers	6	5.3%
Chromosomal abnormalities in the family	2	1.8%
Others	11	9.6%

**Figure 2.**
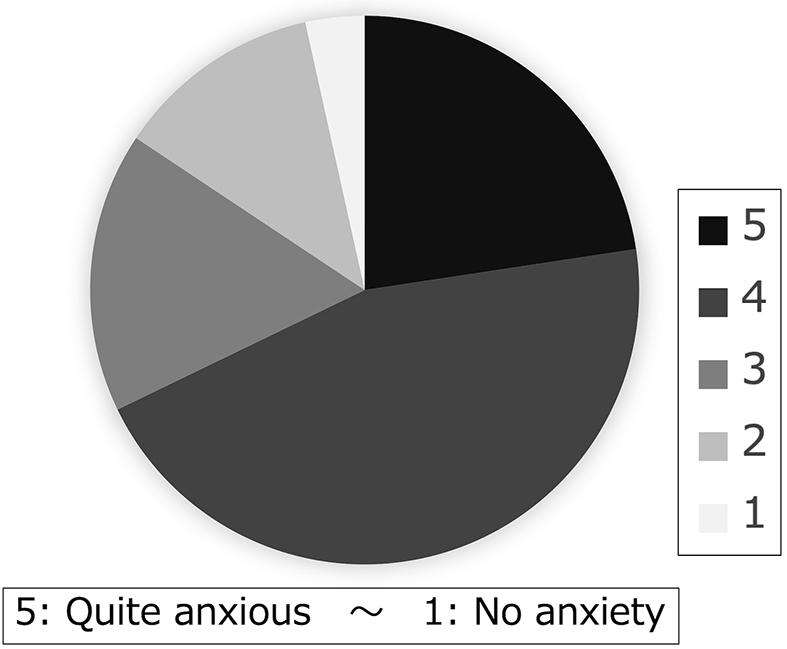
A survey administered after the completion of pre-noninvasive prenatal testing genetic counseling: Precounseling state of mind. More than half of the respondents felt anxious about noninvasive prenatal testing.

**Figure 3.**
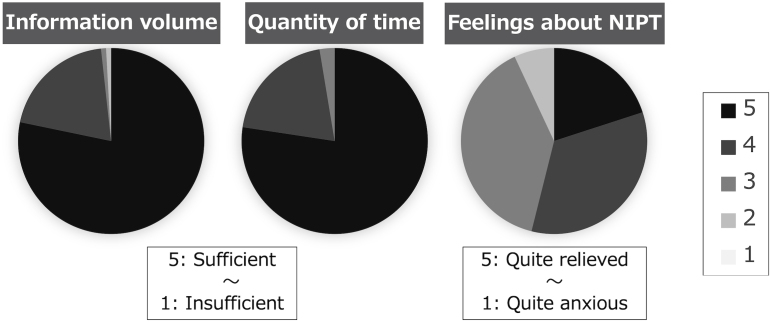
The survey was administered after completing pre-noninvasive prenatal testing genetic counseling: Evaluation of genetic counseling. The level of satisfaction with genetic counseling was generally high. More than half of the respondents felt relieved about noninvasive prenatal testing.

In the survey after initial counseling, 23% of the patients indicated that they were quite anxious, while 45% were fairly anxious before counseling (Figure 2). Regarding the amount of information on genetic counseling, 78% found it sufficient, whereas 20% indicated it was fairly sufficient. Concerning the amount of time for genetic counseling, 77% of the participants have considered the time adequate, while 20% found it fairly adequate. As for their feelings after genetic counseling, 20% felt quite relieved, 34% felt fairly relieved, 39% felt the same, and 7% felt a little anxious (Figure 3).

**Figure 4.**
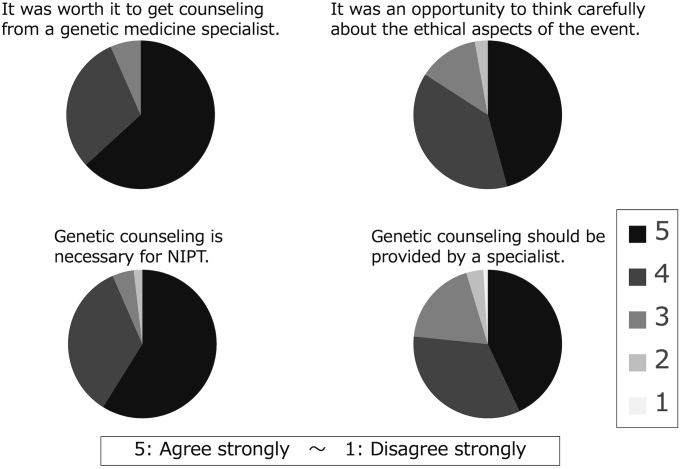
The survey after explanation of the test results (about genetic counseling). Many respondents felt genetic counseling was crucial.

When asked if genetic counseling is necessary for NIPT, 55% strongly agreed, and 32% agreed. Upon asking patients if they could think carefully about the ethical aspects of the procedure through genetic counseling, 43% strongly agreed, and 36% agreed (Figure 4).

**Figure 5.**
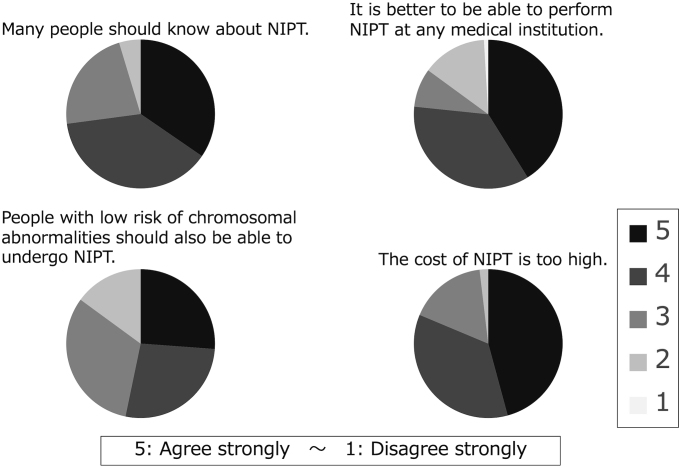
The survey after explanation of test results (about noninvasive prenatal testing). Respondents tended to feel uncomfortable about noninvasive prenatal testing.

When the patients were asked if more people should know about NIPT, 32% strongly agreed, whereas 36% agreed. Regarding whether it would be better if the test could be easily conducted at any medical facility, 38% strongly agreed, and 33% agreed; when asked whether patients thought NIPT is expensive, 43% strongly agreed, and 33% agreed (Figure 5).

Out of all the patients, 5.2% canceled the NIPT examination after preNIPT genetic counseling, 80% thought that genetic counseling was necessary for NIPT, and most patients thought that the environment in which NIPT was conducted should be made more conducive.

In summary, the majority of respondents reported that genetic counseling reduced their anxiety. Moreover, 87% of respondents reported that genetic counseling was necessary. The majority of respondents have requested an increase in the number of facilities where NIPT is available.

## Discussion

NIPT was launched in Japan in April 2013. Over the past 3 years, 52,490 people have received genetic counseling for NIPT, whereas 48,695 people have undergone NIPT ^(4)^. This means that 7.2% canceled after preNIPT genetic counseling, and the 5.2% cancelation in this present study is comparable to this result. Genetic counseling involves an increase in knowledge and a decrease in decisional conflict ^(5)^, and client needs were likely clarified by explaining chromosomes and NIPT characteristics, which may have led to the cancelation of NIPT. This suggests that preNIPT genetic counseling can have the effect of making clients aware of their true needs. This indicates that the importance of genetic counseling was well understood, and yet there was still a need to ease the examination environment. It was thought that this reflected opinions specific to rural areas, where access to examination facilities is difficult.

In addition, several participants believed that preNIPT genetic counseling was necessary. After genetic counseling, most participants felt relieved about their feelings toward NIPT. Genetic counseling before prenatal testing has been shown to reduce anxiety ^(6)^, and comparable results were obtained in this current study. Moreover, for many patients, genetic counseling provided an opportunity to think about bioethics. As long as we are searching for chromosomal abnormalities, we cannot ignore the issue of bioethics. Therefore, genetic counseling is an important opportunity to confront ethics.

It is thus noteworthy that there were calls for easing the current NIPT inclusion criteria and costs. Currently, NIPT is available only at Akita University Hospital in Akita prefecture. Therefore, pregnant women living far away from our hospital have to bear the burden of transportation and costs, implying that not everyone has equal access to the test. As a result, facilities in rural areas may not be actively promoting NIPT. The view of pregnant women on prenatal diagnosis is changing with the passage of time and the advancement of medicine. We thus need to continue paying attention to the needs of pregnant women and their families. The Internet was the most common source of information about NIPT in this study, but information from medical staff and acquaintances was also common. This provision of information from pregnant women’s surroundings may be even more common in urban areas. Regardless of where they live, healthcare providers should support pregnant women to have correct knowledge about prenatal testing.

In recent years, there has been an increase in NIPT use by unlicensed facilities in Japan. The quality of genetic counseling at unlicensed facilities is not assured, and the actual status of testing remains to be clear. This is especially problematic when the NIPT results were not negative. Problems with interpretation and explanation of positive or pending results, and followup have been frequently reported ^(4)^. It goes without saying that it is necessary to understand the characteristics and limitations of the test before undergoing the test, as well as the process to follow if the result is positive. The person providing genetic counseling should be knowledgeable on clinical genetics and have extensive experience in treating the disease in question. In the event of a positive result, a followup system that allows for treatment at an appropriate facility should be assured. The need for NIPT is expected to increase in the future, and discussions are underway on genetic counseling to be provided ^(7)^. Therefore, it is necessary to consider a genetic counseling system that considers both medical resources and the needs of pregnant women.

This survey was subject to limitations. First, being a questionnaire study on pregnant women who have considered undergoing NIPT, this study does not reflect the opinions of all pregnant women. Second, we did not examine the accessibility of the testing facility and where the patients reside. Therefore, it is unclear how accessibility affects patient thoughts on NIPT. Third, this study was not conducted on pregnant women living in urban areas, so comparisons between rural and urban areas cannot be made.

## Article Information

### Conflicts of Interest

None

### Acknowledgement

We would like to express our sincere gratitude to all the pregnant women and their families who participated in the survey. We would like to thank Editage (www.editage.com) for English language editing.

### Author Contributions

YO, HM, AN, IT, AS, TT, and YT were involved in study design and data interpretation.

YO, HM, AF, RN, AN, IT, TT, and YT were involved in the data analysis.

All authors revised the manuscript, approved the manuscript to be published, and agree to be accountable for all aspects of the work in ensuring that questions related to the accuracy or integrity of any part of the word are appropriately investigated and resolved.

### Approval by Institutional Review Board (IRB)

Ethics Review Committee of Akita University School of Medicine (Reception number: 1432).
